# Book Review: Science Fictions: Exposing Fraud, Bias, Negligence and Hype in Science

**DOI:** 10.3389/fpsyg.2021.666441

**Published:** 2021-04-22

**Authors:** Sally A. Larsen

**Affiliations:** Department of Psychology, University of New England, Armidale, NSW, Australia

**Keywords:** replication crisis, scientific method, book review, popular science book, open science

To the outside observer, scientific research may appear a mass of contradictions: each new study claims amazing results, and each subsequent one overrules the last. It is no surprise, therefore, that scientific skepticism appears to be on the rise in many pockets of society (Hornsey, 2020). Into this atmosphere of public uncertainty comes *Science Fictions: Exposing Fraud, Bias, Negligence and Hype in Science*, by Stuart Ritchie, an insider's view of the troubles which plague the modern scientific enterprise. Ritchie, himself a PhD-trained Psychologist and lecturer at the King's College London, presents a call-to-arms for scientists to start repairing a series of endemic problems which, he claims, permeate all areas of modern scientific practice.

*Science Fictions* begins with serious accusations for anyone who's worked as a research scientist over the past few decades: “scientific literature [is] full of untrustworthy, unreliable, unreplicable studies that often do more to confuse than enlighten” (p. 24). To be clear, Ritchie's aim is not to undermine the scientific method: “I come to praise science, not to bury it; this book is anything but an attack on science itself, or on its methods. Rather it is a defense of those methods, and of scientific principles more generally, against the way science is currently practiced” (p. 9–10). It's not the scientific method that's the problem, it's the people involved: the scientists. Despite this aim the balance of the book, and the most interesting parts, focuses on the most shocking examples of how wrong things can go. A casual reader might leave the book with an uneasy feeling that the entire world of scientific research rests on foundations of sand, with not much hope of shoring it up.

Each thematic chapter in Part 2, “Faults and Flaws” describes a series of eye-opening examples of where the scientific process has been sent awry by fraud, bias, negligence and hype. For example, the fraud section covers blatant data fabrication published in academic journals as legitimate research; the bias chapter gives an incisive summary of the way publication bias affects the results of meta-analyses; the negligence section reports depressing details on the extent to which published research is replete with statistical errors; and the hype section discusses how popular science books, written by experts in a field, can distort and exaggerate the nature of a scientific finding, distilling down the complexity of an area to a simplified, news-grab message. These reductive versions of complex research findings are those that find their way into the media and reinforce the perception that science can't make up its mind. There's an interesting irony at work here: *Science Fictions* itself capitalizes on hype in order to draw in the reader. The examples given are the worst possible scenarios, those that endanger human lives, muddy the waters of the scientific record, and put careers and money ahead of ethics and incremental progress.

Ritchie goes even further when he proposes that most flawed scientific work is never discovered. In relation to data fabrication: “If this kind of fraud occurs at the very highest levels of science, it suggests that there's much more of it that flies under the radar, in less well-known journals” (p.60). Is this evaluation completely justified? There's no way of knowing, but it seems at least possible that data may be “tweaked” for the purposes of fitting the brief of prestigious journals, so that authors can claim both the status of a groundbreaking finding and a publication in an internationally renowned outlet. The vast majority of scientists working to develop incremental knowledge might be less likely to participate in such schemes.

In a change of tone, the somewhat optimistically titled final chapter, “Fixing Science,” proposes a number of solutions for repairing the damage. These include improving training in statistics, encouraging journals to more readily publish replication studies, and using technological advances to reduce the possibility of unintended errors creeping into analyses. Ritchie also covers the emerging Open Science movement (Foster and Deardorff, 2017), and the practice of preregistering research studies (Nosek et al., 2018). While these suggestions are all worthy of serious consideration, and are already making an impact, Ritchie admits that such solutions are “mainly aimed at addressing the *symptoms* of science's modern maladies, rather than the causes” (p.226). In other words, all the goodwill in the world on the part of individual scientists, or groups of like-minded individuals is unlikely to make a dint in the “perverse” incentive structure that currently exists for scientific research.

There's something of a contradiction in terms in the overall theme that runs through the book. On the one hand Ritchie insists that it is the flaws in human nature that underlie the problems, rather than faults in the scientific method. On the other hand, the notion that the whole enterprise might be rescued via the efforts of those same flawed people seems overly optimistic. *Science Fictions* leaves a reader without a doubt that there are difficult problems that need to be resolved, and it is the responsibility of the scientific community to confront the issues. Nonetheless, it's not entirely clear who Ritchie's intended audience is. Working scientists are likely already well-aware of the problems in the system, but the general reader might not be overly interested in directions for scientists on how to go about clearing up their own backyard. Unfortunately, the book contains few examples of the scientific process working in the way it is supposed to, which might have the opposite effect in the public domain than what is intended. Readers might come away with a greater skepticism of any scientific findings, rather than more faith that the process will discover “the truth” about anything. While the book is clearly an extended variation on a long-running discussion within the scientific literature (e.g., Ioannidis, 2005; Moonesinghe et al., 2007), whether a popular science book is the right forum for this discussion I will leave to the reader to decide.

## Author Contributions

SL conceived the article, and entirely wrote and edited the manuscript.

## Conflict of Interest

The author declares that the research was conducted in the absence of any commercial or financial relationships that could be construed as a potential conflict of interest.
